# Role of Technological Acquisition and R&D Expenditure in Innovative Investment

**DOI:** 10.3389/fpsyg.2022.855295

**Published:** 2022-04-05

**Authors:** Zou Weiyu, Aniza Othman, Tang Guli

**Affiliations:** ^1^Business School, Universiti Teknologi Malaysia, Johor Bharu, Malaysia; ^2^Taihu University of Wuxi, Jiangsu, China

**Keywords:** technological acquisition, research and development, innovation investment, attitude toward digital innovation, technological culture

## Abstract

Despite the mounting importance of digitalization among industries and the corporate sector, the stress on the transformation of business operations is limited, thus creating a gap in the literature. The current study aims at determining the role of technological acquisition and research and development (R&D) expenditure in innovative investment. Technological acquisition and R&D are two crucial indicators of digital innovation. Therefore, to understand this, the current study collected data using a questionnaire survey method from 341 employees of the R&D department in the corporate sector of China. Data analysis was performed using the structural equation modeling (SEM) technique. The software used for the statistical analysis of the data was Smart-PLS. Results of the study showed a significant relationship between the independent variables (technological acquisition and R&D) and the dependent variables (innovative investment and attitude toward digital innovation). The study also found that attitude toward digital innovation among the employees positively and significantly impacted innovative investment. Moreover, attitude toward digital innovation acts as a partial mediator between technological acquisition and innovative investment, and R&D and innovative investment. Furthermore, technological culture significantly moderated the relationship between technological acquisition and innovative investment, but did not moderate the relationship between R&D and innovative investment. Henceforth, to practically imply the present study, it is important to ensure the use of the technology is made common by providing training to the employees so that the technical skills of the employees can be polished and utilized for the betterment of the firm.

## Introduction

Innovation is not dispersed haphazardly across businesses. Instead, it results from strong approaches, investments, and partnerships that businesses constantly seek out and create in the marketplace. Current research examines the causes of innovative investment at the firm level by examining research and development (R&D) expenditures and purchasing technologies for both embodied (machines and tools) or disembodied (software). R&D is defined as “all systematic creative activity conducted to grow the stock of knowledge and to use this stock to create new applications, such as new or improved products (good and services) and procedures (particularly software research)” (Conte and Vivarelli, 2014). Technological advancement has aided the progress of human civilization. Economic and social growth would not have been feasible without significant breakthroughs in this area throughout the period. A study of the innovative process is always a topical issue on both academic and practical aspects since it is a continual activity. The term “innovation” has been used in the literature to describe both the process of generating new goods using new information, technologies, and procedures, along with new or better products themselves.

Individuals, businesses, governments, and the world all take benefit from innovation. From a geographical standpoint, the growth of information and communication technologies, a product of technical innovation, favors the speeding up of the inventive process’s outcomes distribution (Xiaolong et al., 2021). In business practices, workplace organization, or external relations, the conceptual framework for innovation describes innovation as deploying a new or considerably enhanced product or process as a new marketing approach or a new organizational method. The above definition addresses two key points: that the “innovation” process encompasses the technological development of an invention and its entry into the market to end-users through adoption and diffusion, and that the innovation process is iterative, hence, the first introduction of an improved innovation is automatically included. At the global scale, the Green Paper defines innovation as the creation and application of effective new solutions to economic and social issues that meet individual and societal requirements, resulting in changes in global economy sectors (Garcia and Calantone, 2002).

Innovation is the pillar of strength and vitality for businesses. They always begin with a new idea, at least in relation to their competitors. They must continue to innovate, survive, and grow. Because innovation entails predicting market needs and providing quality and ancillary services, efficient organization, and expertise, the technical advancement becomes insufficient to achieve success from this standpoint. Technological innovation is the implementation of creativity that results in innovations. These ideas may be found at various phases of the inventive process, which includes the activities of generating ideas, developing a service or product, and selling it. These three processes entail some sort of investment. Such investment opportunities fall into two categories: (1) offensive strategic investments which aim to keep the organization at the technological forefront in its field of interest and increase market share through traditional managerial approaches, and (2) defensive strategic investments which correspond to high-risk bets on the future, through which companies implement projects in similar locations to their competitors, but at various melodic lines (Zirra, 2020).

Firms now work in a quick dynamic business environment, and they should develop as well or their procedures will become antiquated, with their goods (or services) becoming less competitive. Customers’ needs, tastes, and expectations of these companies’ products change throughout time. Owners and managers of businesses must make judgments in an unpredictable environment while making the most out of scarce funds. Companies must safeguard and maintain technology to remain competitive, and businesses must obtain knowledge on future technologies while designing asset repair and operation programs. The expense of maintaining a company’s technology rises as it ages and deteriorates. Because of technological development, both new capital and maintenance costs fluctuate during the life of a fixed asset (Nguyen et al., 2017). According to the literature, the ideal asset (technology) lifespan is usually shorter when new capital costs fall faster than the slow falls of maintenance costs, or when both new investment and operational costs fall at the same rate (Zirra, 2020).

Organizations must budget for the upkeep of existing hardware and the procurement of new technology. Furthermore, access to formal finance remains a significant barrier for small and medium-sized businesses, particularly in emerging nations. Accessibility to conventional sources of capital, such as banks, has an impact on a company’s export success. The impact of severe financial limitations on business owners and management differs (Yatsenko and Hritonenko, 2009; Quartey et al., 2017). In certain circumstances, a shortage of financing causes business owners and managers to have a negative attitude toward conventional sources of finance. Because of entrepreneurs’ poor perceptions of formal finance, some have turned to inefficient informal lending sources, particularly when faced with severe credit limits (Tolba et al., 2014; Heshmati, 2015; Archer et al., 2020). Nonetheless, because of their key decision-making position, the impression of business owner and managers is critical. To handle entrepreneurial finance difficulties, the government’s policy framework for funding or assisting small and medium businesses is also critical (Mallinguh et al., 2020).

Acquisitions have an unmistakable effect on a company’s ability to innovate. The assessment is critical for organizational learning and innovation as it helps to define how companies receive and utilize external information. Innovativeness, according to certain theories of technological progress, is the result of an increased knowledge base (Griliches, 1990; Ahuja and Katila, 2001). Acquisitions might be driven by a desire to acquire access to markets, penetrate new markets, or gain financial synergies or market strength. The impact of acquisitions on a company’s innovation output may be defined in terms of the technological inputs that the acquisition provides. Acquisitions can have two different effects on eventual innovation capability. To begin, the acquisition of some other business might be thought of as the absorption of the acquired firm’s knowledge base (Ahuja and Katila, 2001; Fartash et al., 2018). It is common knowledge that innovation has an impact on a region’s economic development and growth. Few experts believe that properly and efficiently embracing current technology is a critical component of maintaining growth and development. As a result, R&D spending, which serves to encourage innovation, is a critical component of attaining long-term economic growth. Economic academics say that innovation stimulates economic development on a global scale (Callegati et al., 2005; Hu, 2015; Huggins and Thompson, 2015). In most industrialized nations across the globe, technological transformation is strongly linked to economic growth, and these industrial transformations are linked to high levels of R&D spending and innovation (Akcali and Sismanoglu, 2015; Itoandon, 2016).

According to Daft, there are two sorts of organizational dimensions: structural and contextual. Technology, which refers to the tools, processes, and activities needed to convert inputs into outputs, is one of the contextual dimensions. Organizational technology encompasses topics like flexible manufacturing, modern information systems, the Internet, and how the company creates the products and services it gives to consumers. The underlying collection of important values, beliefs, understandings, and conventions held by employees is a company’s culture (another contextual component of the organization). Culture is the glue that holds an organization’s members together, and it can refer to ethical conduct, employee devotion, efficiency, or customer service (Daft, 1992; Wyrwicka, 2011).

Technological culture, according to Wolk, is a logical, artistic, and, from a social standpoint, positive attitude toward the use of technology to improve a society’s quality of economic, social, and spiritual daily life in line with the level of technical (technological) advancement. Furmanek used the term “technological culture” to characterize people’s ability to make proper use of technology in their surroundings to improve their quality of life. Technological culture is manifested in the creators’ constant and positive attitudes toward technology and technical expertise, but it is primarily manifested in the ethical behaviors and acts of diverse technological circumstances (Owen-Jackson, 2002; Combi, 2016). Technical culture manifests itself in relatively long-term activities and good ethical human attitudes, allowing for the correct application of existing technologies and the development of new technical solutions to enhance the effectiveness of life’s cooperative processes (Leidner and Kayworth, 2006).

With the advancement in technology and its acquisition, several researchers have found a role of technological acquisition and spending in R&D toward achieving the innovation. There was a gap in finding the role of technological acquisition and R&D on innovative investments. Although much research has been conducted to find the combined effect of all these in organizational performance, no research has so far been conducted to find the role of technological acquisition and R&D expenditure on innovative investment, itself, as determiner. Several researchers have found the impact of technological transformation on developing a culture, leaving a gap in examining the role of technological culture itself as a moderating factor between technological acquisition, R&D expenditure, and innovative investment (Khin and Ho, 2018). Similarly, no prior research has yet been reported on attitudes of digital innovation, mediating the function of innovative investments in any dimension. It raised several research questions like “how the technological acquisition and R&D expenditures could direct the innovative investments?,” “How could technological culture facilitate the processes of technological acquisition and R&D expenditures toward innovative investments?,” and how the attitude toward digital innovation could regulate the functioning of innovative investments?

Addressing these research questions and filling the gaps in research, we devised the following research with certain objectives, such as to find the role of technological acquisition and R&D expenditure on innovative investments and attitude toward digital innovation, to evaluate the mediating role of the technological role between technological acquisition, R&D expenditure, and innovative investments and to explore the moderating role of attitudes toward digital innovation in the process of innovative investments.

## Review of Literature and Hypotheses Development

This research was conducted in China and the respondents were from R&D departments of different organizations. The research evaluated the role of technological acquisitions and R&D expenditures in innovative investment and attitude toward digital innovation. Moreover, technological culture that mediated the relationships and moderating effects of attitudes toward digital innovation were also tested. The framework of this study was based on the following theories.

### Investment Theory of Creativity

According to the investment theory of creativity, which was created in partnership with Todd Lubart, creativity is mostly a decision. In the domain of ideas, it is specifically a decision to purchase low and sell high. Creative individuals, like excellent investors, produce ideas that are fresh and are now, sometimes, a little ludicrous. The creative is “buying low” in a figurative sense. The innovative folks then “sell high,” earning the riches of their brilliant concept before moving on to the next controversial idea once their ideas have garnered some acceptability. People who are naturally creative prefer to stand out from the crowd. Individuals don’t want to think or act in the same way that others do. Instead, they prefer to veer out in their own route, attempting to come up with fresh and valuable ideas. As a result, the most significant impediment to creativity is, frequently, one’s own mental limits rather than restrictions imposed by others. Nevertheless, these constraints may arise as a result of factors in the spread and socialization processes, making it difficult to determine if restraints on creativity are imposed internally or outside (Sternberg and Lubart, 1991).

In almost the same manner that investment is a decision, so is creativity. Individuals aren’t born with the ability to be creative or uncreative. Instead, they build a set of life attitudes that distinguish individuals who are prepared to pursue their own path. Readiness to (a) reinterpret difficulties in novel methods, (b) follow to ensure risks, (c) “advertise” theories that some may not at first recognize, (d) keep faith in the face of challenges, and (e) investigate as to if their own preconceived notions are interfering with their creative process are examples of such attitudes toward life. Such attitudes may be taught to pupils and instilled in them through training that encourages them to think for themselves. Ability, expertise, cognitive patterns, personal characteristics, motivation, particularly intrinsically motivated, and surroundings are all factors that influence innovation (Lubart and Sternberg, 1995; Sternberg, 2012).

An individual may possess the creative talent to allow for creativity, but without the desire to take reasonable risks or an environment that offers at least basic support for creativity, that person’s potential creativity may be stifled. It is therefore critical, particularly in schools, to create an environment that encourages creativity not just in words, but also in action. At the same time, while a person can have a creative mindset, he or she may not be able to fulfill their full creative potential without the abilities of creativity, such as seeking ways to reconcile contradictory ideas and using dialectical reasoning (Sternberg and Lubart, 1993; Sternberg et al., 1997; Sternberg, 2012). So, this theory provided the basis for investment in innovation, and we utilized the theoretical support for innovative investment in the model.

### Diffusion of Innovation Theory

One of the earliest social science ideas is E.M. Rogers’ Diffusion of Innovation (DOI) Theory, which he created in 1962. It was first used in marketing to describe how such an industry develops traction and disperses (or travels) through a population or social system over time. The eventual effect of this dissemination is that individuals embrace a new concept, habit, or product as part of a social structure. Adoption entails someone doing something unique from what they formerly did (e.g., purchasing or using the new product, acquiring and performing a new behavior, etc.). Adoption depends on the perception of the concept, behavior, or commodity as novel or unique. Diffusion is conceivable as a result of this (Rogers and Singhal, 1996). Diffusion, according to Rogers, is the process through which an invention is disseminated among the members of a social system over time. The spread of innovations hypothesis has a wide range of sources that span different fields (Rogers et al., 2019). Numerous researchers have incorporated broad diffusion theories of technological innovation based on Rogers’ theory to adopt and diffuse an innovation technology in higher education institutions at both the macro and micro levels. Rogers’ theory has been frequently employed in this respect to explain why the acquisition and spread of innovation differ among societies.

Diffusion, as per Rogers, is “the process by which an invention gets disseminated through particular channels among the members of a social system over time,” where the “innovation” might be anything that the adopters perceive as novel. It is the most often cited theory in the subject of innovation dissemination and is comprised of four key factors of spread: invention, channels of communication, timing, and the social structure. Many investigations, hypotheses, and models on the subject of diffusion have been based on this principle. Rogers’ idea has been utilized in a number of researches to explain why some people absorb technology innovation while others do not. In this regard, Rogers claims that everyone follows a normal distribution when it comes to technology adoption and diffusion. This suggests that there is a bias toward the necessity to disseminate technology without taking into account the repercussions of doing so (Sahin, 2006). Change initiatives are focused on quick adoption and dissemination to get instant outcomes without contemplating the ramifications on the social structure. Rogers’ social system has two basic keywords or concepts: adaptability with technology, and tech enthusiasts (Dintoe, 2019). This theory provided the basis for evaluating the role of technological acquisitions.

### New Growth Theory

The New Growth Theory contains two key points and is a way of looking at the economy. For starters, it considers technology advancement to be a byproduct of business growth. Previously, technology was assumed to be a fixed result of non-market causes. Since it incorporates technology into a theory of how trade works, the New Growth Theory is frequently referred to as the “endogenous” growth theory. Second, according to the New Growth Theory, knowledge and technology, unlike physical goods, are defined by rising returns which drive the growth process. Romer is attributed with popularizing New Growth Idea, however, the theory itself is nothing new, as Romer himself points out. The primary idea underlying the New Growth Theory is that new information or technology brings greater yields. Standard economic theories are built on the concept of decreasing returns. It states that when you raise the output of something (e.g., a farmland, a business, an entire economy), adding more inputs (e.g., labor effort, equipment, land) produces less return than adding the very last unit of a product. Declining returns are essential because they lead to greater variable costs (i.e., at some point, the cost of generating one more unit of output exceeds the cost of creating the previous unit of a product) (Romer and Griliches, 1993; Romer, 1994, 1996; Cortright, 2001).

### Relationship of Technological Acquisition With Innovative Investment and Digital Innovation

The governments and scholars have generally verified the significance of technology as a source of sustainable competitive advantage for sectors, particularly in industrial industries (Appolloni et al., 2022). To achieve this competitive edge, it is critical to understand both the technological tools and the best approaches for enterprises to manage technology (Gastaldi et al., 2022). Technology strategy is characterized as the successful identification, selection, acquisition, development, exploitation, and preservation of technologies (including product, procedure, and even infrastructural) required to achieve, maintain, and perform in accordance with the strategic goals of the company (Tsilionis and Wautelet, 2022). Acquisitions have an evident influence on organizations’ innovation performance (Strobl et al., 2022). This evaluation is critical for organizational learning and innovation because it clarifies how companies receive and use knowledge resources (Andrade et al., 2022). Some theories of technological evolution suggest that an increased knowledge base leads to increased innovativeness (Pan et al., 2022).

Acquisitions might be driven by a desire to acquire access to distribution networks, gain access to new markets, or obtain financial synergies or market strength (Wang et al., 2022). The influence of acquisitions on the firm’s innovation output may be viewed in the context of the acquisition’s new advanced technologies (Renko et al., 2022). Acquisitions can have an impact on subsequent productivity and creativity via two different approaches (Francini et al., 2022). Furthermore, an acquisition of another firm may be considered as an absorption of the acquired firm’s level of understanding into the knowledge base of the acquired company (Ahluwalia and Kassicieh, 2022). Technology management has been one of the most essential organizational concerns to deal with a dynamic market scenario, and systematic performance management, as a source of sustainable competitive advantage, is critical for many businesses. Based on the theoretical support for technological acquisition in achieving innovativeness in the corporate sector, we hypothesized the following.

H_1_. *Technological acquisition has a positive impact on innovative investment.*
H_2_. *Technological acquisition has a positive impact on attitude toward digital innovation.*

### Relationship of R&D Expenditure With Innovative Investment and Digital Innovation

Research and development (R&D) investment is critical for businesses that wish to generate new knowledge, increase their capacity to invent, and conduct technical innovation activities, particularly in today’s dynamic economy (Keinz and Marhold, 2021). R&D investment involves the use of numerous types of tangible or intangible resources, such as financial resources, technological resources, and professional R&D staff (Cao et al., 2021). According to the resource-based view (RBV), spending substantial amounts of precious and uncommon resources is beneficial to process efficiency and the launch of new goods (Xiao et al., 2021). Digital innovation initiatives that result in the introduction of new digital products or services on a bigger scale must be differentiated (Komninos et al., 2021). There are various techniques that are available today for comprehending the process of developing new knowledge and information, often known as digital innovation activity (Hadjielias et al., 2021). It was hypothesized that the significance of R&D expenditure toward innovation would produce significant results in devising the investments for innovation. Such type of relationships could be a helping hand for future researchers. Hence, we identified the following as hypotheses.

H_3_. *R*&*D expenditure has a positive impact on innovative investment.*
H_4_. *R*&*D expenditure has a positive impact on attitude toward digital innovation.*

### Impact of Attitude Toward Digital Innovation and Its Mediating Role

According to the researchers, attitudes may be developed internally and/or persuaded with enough effort and time (Shrum et al., 2012). They defined attitudes as a judgment process influenced by emotions, opinions, and actions which they referred to as internal attitudes (Iveroth and Bengtsson, 2014). The theme of a story is sometimes referred to as an internal attitude, since it involves personal gains for personal ideas (Jepson et al., 2012). Externally, persuasion based on credibility, sympathy, and logic will refine and impose arguments on people’s attitudes, affecting their moral duty (Higgins and Walker, 2012). In addition to the antecedents, external influences, such as eco-centrism and altruism, are thought to impact attitudes (Abu Bakar et al., 2020). Those working on sustainability studies have a diverse range of views on technology. These people range from those who see innovation efficiency gains as the feature to bring up sustainability issues to those who see them as the root of the problem, believing in that increasing absolute resource consumption through rebound effects and speeding up the disruption of natural ecosystem cycles by introducing ever more alien substances. There are countless and, at first glance, perplexing permutations and combinations of attitudes in between these two situations (Ehlers and Kerschner, 2013).

Translating information into economic activity is what innovation is all about. It is a multi-source activity of discovering, understanding, and using new technologies and processes. Digital innovation is the invention and implementation of novel products and services, while digitalization is the result of several digital innovations resulting in novel actors (and actor groupings), structures, practices, values, and religious views that change, try to intimidate, replace, or supplement existing rules of the game within organizations and fields. The application of digital technology in a wide range of advancements is referred to as digital innovation. The term “digital” refers to the conversion of mostly analog information into the binary code accepted by computers. Malleability (e.g., re-programmability), similarity (e.g., standardized software languages), and generalizability of the findings (e.g., ease of converting digital representations of any object) are at the core of technologies that mesh digital and, often, physical materiality, thereby enabling and constraining, but also intertwining, human action. Although attitudes toward adopting e-learning have been tested as mediators before (Altawalbeh et al., 2019), no study has found the attitude toward digital innovation as a mediator in any set of research. Hence, it was proposed that attitudes toward digital innovation could lead to the following hypotheses. Meanwhile, the mediating role of digital innovation has also been studied before and presented significant results (Khin and Ho, 2019; Yasa et al., 2019).

H_5_. *Attitude toward digital innovation has a positive impact on innovative investment.*
H_6_. *Attitude toward digital innovation mediates the relationship of technological acquisition and innovative investment.*
H_7_. *Attitude toward digital innovation mediates the relationship of R*&*D expenditure and innovative investment.*

### Moderating Role of Technological Culture

Since the early 1980s, the culture of an organization has been a popular issue. Corporate culture has been recognized as a key component of organizational performance throughout the previous two decades (Martín-de Castro et al., 2013). It is characterized as a deeper level of underlying principles, assumptions, and beliefs held by members of an organization. More specifically, innovation culture refers to organizational members’ shared similar values, ideas, and assumptions that may help the creative innovation process (Stanley and Swann, 2021). When an organization’s culture or environment stimulates employees’ ability to innovate, tolerates risk, and promotes personal growth and development, the organization’s culture is referred to as an innovation culture.

Technological culture is a logical, artistic, and a positive attitude toward the use of technology to improve a society’s quality of economic, social, and spiritual daily life in line with the level of technical (technological) advancement (Rosenzweig, 2022). Technological culture is manifested in the creators’ constant and positive attitudes toward technology and technical expertise, but it is primarily manifested in the ethical behaviors and acts of diverse technological circumstances (Owen-Jackson, 2002; Combi, 2016). Technical culture manifests itself in relatively long-term activities and good ethical human attitudes, allowing for the correct application of existing technologies and the development of new technical solutions to enhance the effectiveness of life’s cooperative processes (Leidner and Kayworth, 2006). No prior study has evaluated the role of technological culture as a moderator between relationships or processes of innovative investment. The use of technological culture as a moderator was suggested by Khin and Ho (2018). This helped us to evaluate the following hypotheses.

H_8_. *Technological culture moderates the relationship between R&D and attitude toward digital innovation.*
H_9_. *Technological culture moderates the relationship between R&D and attitude toward digital innovation.*

**FIGURE 1:**
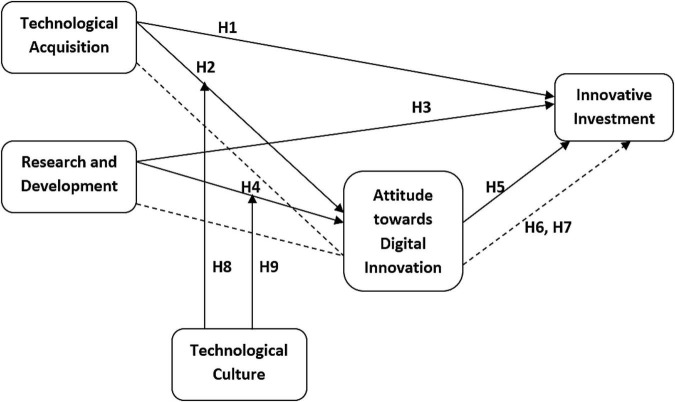
Conceptual framework.

The following conceptual model (Figure 1) has been formed based on the above literature and hypothesis.

## Methodology

A quantitative research design was implied in the present study by expressing the variables of the study in numbers and analyzing the relationships hypothesized in the study through statistical methods. A deductive approach was used to confirm the hypotheses of the study that were established to investigate the effect of independent variables on the dependent variables. The research philosophy followed in this study was post-positivism as the present study conducted is a causal study in which the effects of the specified independent variables were studied particularly on the dependent variables. This study was directed toward measuring the attitudes and behaviors of the respondents in the prescribed scenario. Therefore, applied and explanatory research has been incorporated to explain the causal effects among the variables. This study was based on the primary data that Was collected directly from the respondents by the researchers through the questionnaire survey method. Since the present study intended to identify the patterns, characteristics, and causal relationships among the variables, it formed the basis for a descriptive type of research. The current research study aimed to test hypotheses developed after a detailed review of the literature regarding the relevant variables used.

Data collection was carried out with the help of a self-administered survey to acquire quantitative data from the study participants. The target population of the study included the employees of the research and development department in the corporate sector. The sample was selected on the basis of convenience sampling. This technique was beneficial as data from readily and conveniently available respondents could be obtained. In addition, this method is less expensive and less time-consuming (Stratton, 2021). The questionnaires were distributed among the employees working in the R&D departments of the corporate sector. The survey method was used to understand the general patterns, opinions, and characteristics of the targeted groups of people. Prior permissions were sought from the corresponding organizations to avoid any inconvenience during the process. The researcher had approached the respondents in person, and the questionnaires were distributed among the respondents who were willing to participate in the research. However, during the post-COVID situation, when people were generally terrified of getting in contact with the deadly virus, they tended to stay away from each other (Gabriel and Aguinis, 2021). People were likely to maintain social distance and were not very much willing to accomplish the survey. Therefore, some of the questionnaires were left with the human resource managers to get filled when the respondents were ready to accomplish the survey. Those questionnaires were collected after 3 weeks. The ethical consideration of the study had been practiced by ensuring the respondent’s confidentiality of their particulars. Biases in the research due to convenience sampling were reduced by using this approach and research design. Data analysis was conducted after the data was collected from the respondents. The sample size of the present study was 341. The unit of analysis for the present study was the employees of the R&D department in the corporate sector of China.

### Instrument Development

The current study used a questionnaire as the survey instrument. The items for each variable were present in the questionnaire. There were five variables in the present study, i.e., two independent variables that were about technological acquisition and R&D expenditure, one dependent variable that was about innovative investment, one mediator that was about the attitude toward digital innovation, and one moderator that was about technological culture. The variables had been operationalized based on the past studies (Ashok et al., 2016; Fartash et al., 2018; Hameed et al., 2018; Khin and Ho, 2019). Thus, the items for each variable have been adapted from past studies based on the operational definitions used in the study. The items for each construct were adopted from prior studies that took place in similar settings. The scale for the technological acquisition was adopted from Fartash et al. (2018), which consisted of 5-items. The scale for R&D expenditure was adopted from Hameed et al. (2018), which consisted of 5-items. The scale for innovative investment was adopted from Ashok et al. (2016), which also consisted of 5-items. The scale for attitude toward digital innovation was adopted from Khin and Ho (2019), which consisted of 6-items. Lastly, the scale for technological culture was also adopted from Khin and Ho (2019), which also consisted of 6-items. The present study used a 5-point Likert scale to obtain the quantitative data from the respondents. The responses were graded with 1 being the highest degree of disagreement and 5 being the highest degree of agreement.

### Demographics Details

The first part of the questionnaire consisted of the demographic particulars of the respondents. It included the questions about their gender, age, education, and the tenure of the respondents for working in the R&D departments. The total number of employees who took part in the study was 341, out of which 56.30% were male and 43.79% were female. The employees between the age bracket of 20 and 30 years were 34.31%, the employees who were between 31 and 40 years were 31.96%, the employees between the age of 41 and 50 years were 19.06%, and the employees who had an age above 50 years were 14.66%. Moreover, the respondents who had bachelor’s degrees were 54.84%, the respondents with master’s degrees were 29.33%, and the respondents who possessed a Ph.D. or some other degree were 15.84%. The employees with an organizational tenure of less than 1 year were 28.74%, the precipitation of the respondents with an organizational tenure between 1 and 3 years were 33.72%, the respondents who had an organizational tenure between 4 and 6 years were 21.99%, and the participants with an organizational tenure of more than 6 years were 15.54%. The details of the demographics can be seen in Table 1.

**TABLE 1:** Demographics analysis.

Demographics	Frequency	Percentage
**Gender**		
Male	192	56.30%
Female	149	43.79%
**Age (years)**		
20–30	117	34.31%
31–40	109	31.96%
41–50	65	19.06%
Above 50	50	14.66%
**Education**		
Bachelors	187	54.84%
Masters	100	29.33%
Ph.D. and others	54	15.84%
**Organizational tenure (years)**		
Less than 1	98	28.74%
1–3	115	33.72%
4–6	75	21.99%
More than 6	53	15.54%

*N = 341.*

## Data Analysis and Results

The structural equation modeling (SEM) technique was applied to analyze the data that was obtained from the respondents. This technique required the use of Smart-PLS 3.3.3 software. There are two stages for data analysis using this software, i.e., measurement model and structural model. In the measurement model, the validity of the data was examined using factor loading, average variance extracted (AVE), Hetero Train Mono Trait (HTMT), and Fornell and Larcker tests, while the reliability of the data was analyzed using Cronbach alpha and composite reliabilities. The structural model was examined to confirm the hypotheses of the study using *p*-value, *t*-statistics, sample means, and standard deviation.

### Measurement Model

**FIGURE 2:**
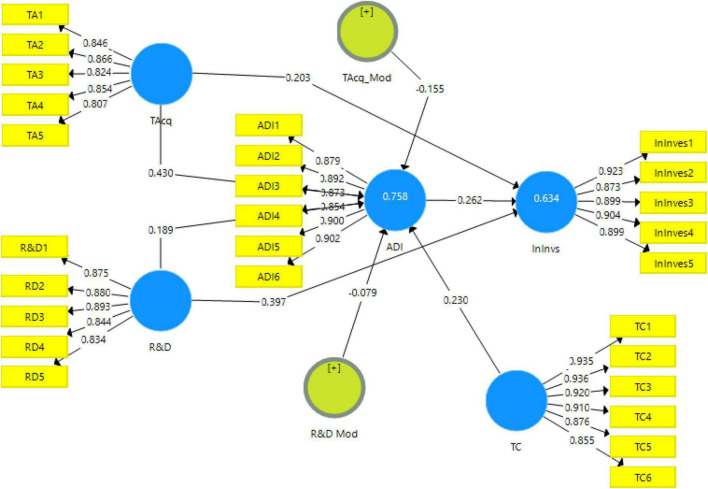
Output of measurement model. TAcq, Technological Acquisition; R&D, Research and Development; InInvs, Innovation Investment; ADI, Attitude toward Digital Innovation; TC, Technological Culture.

The output of measurement model algorithm (see Figure 2) presents the degree of contribution of independent variables onto dependent variables of the study.

Each item’s factor loadings along with Cronbach alpha, composite reliability, and AVE values are in the table below. Multiple items are required to measure variable and factor loadings to help explain how much each item contributes toward a variable (Chan and Lay, 2018). Factor loadings are divided into three categories. Factor loadings less than 0.60 are considered undesirable, factor loadings greater than 0.60 are considered fair, and factor loadings greater than 0.70 are considered highly desirable (Dash and Paul, 2021). Thus, the current study set the benchmark value of 0.6 for the factor loadings and the result (see Table 2 below) shows that the factor loading for each item is more than 0.60.

**TABLE 2:** Factor loadings, Cronbach Alpha, composite reliability, and average variance extracted (AVE).

Variables	Factor loadings		Cronbach alpha	Composite reliability	AVE
Technological acquisition	TA1	0.846			
	TA2	0.866			
	TA3	0.824	0.896	0.923	0.705
	TA4	0.854			
	TA5	0.807			
Research and development	RD1	0.875			
	RD2	0.880			
	RD3	0.893	0.916	0.937	0.749
	RD4	0.844			
	RD5	0.834			
Innovation investment	InInvs1	0.923			
	InInvs2	0.873			
	InInvs3	0.899	0.941	0.955	0.809
	InInvs4	0.904			
	InInvs5	0.899			
Attitude toward digital innovation	ADI1	0.879			
	ADI2	0.892			
	ADI3	0.873	0.944	0.955	0.781
	ADI4	0.854			
	ADI5	0.900			
	ADI6	0.902			
Technological culture	TC1	0.935			
	TC2	0.936			
	TC3	0.920	0.956	0.965	0.820
	TC4	0.910			
	TC5	0.876			
	TC6	0.855			

*N = 341.*

Cronbach alpha determines the internal consistency between the items of a particular variable. Its value should be greater than 0.70 (Hair et al., 2017). The Cronbach alpha for each construct of the presents study is greater than 0.70 (see Table 2), which indicates that every construct is highly reliable. Composite reliability values are classified into three categories, i.e., a value of 0.60 indicates fair reliability, a value between 0.60 and 0.70 indicates satisfactory reliability, and a value between 0.70 and 0.90 indicates highly satisfactory reliability (Peterson and Kim, 2013). The result shows that the composite reliability of each construct is greater than 0.60 (see Table 2 below), indicating satisfactory reliability of the constructs. Table 2 below also shows the result for AVE, which came out to be greater than 0.50. This indicates that convergent validity is present.

The presence of the difference between the variables of the study is determined through the discriminant validity. This validity is tested with the help of the HTMT Ratio and the Fornell and Larker Criterion (Franke and Sarstedt, 2019). The value of the HTMT ratio of less than 0.90 indicates the existence of discriminant validity (Franke and Sarstedt, 2019). The results for the HTMT ratio for each variable under study is less than 0.90 (see Table 3 below), indicating discriminant validity between constructs. Likewise, considering the Fornell and Larker Criterion for determining the discriminant validity of a variable, the top value of every column should be higher than the corresponding values (values below) for a particular variable (Franke and Sarstedt, 2019).

**TABLE 3:** Hetero trait mono trait (HTMT) ratio.

	ADI	InInvs	R&D	TAcq	TC
ADI					
InInvs	0.771				
R&D	0.803	0.804			
TAcq	0.888	0.779	0.832		
TC	0.554	0.435	0.445	0.533	

*TA, Technological Acquisition; RD, Research and Development; InInvs, Innovation Investment; ADI, Attitude toward Digital Innovation; TC, Technological Culture.*

Table 4 shows the result for Fornell and Larker Criterion. It can be seen that the top value of every column should be higher than the corresponding values (values below) for each variable under study. Furthermore, the R-square (*R*^2^) value of the mediating variable came out to be 75.6%, while the R-square (*R*^2^) value of the dependent variable is shown as 63.4%.

**TABLE 4:** Fornell and larcker criteria.

	ADI	InInvs	R&D	TAcq	TC
ADI	0.883				
InInvs	0.727	0.900			
R&D	0.748	0.748	0.865		
TAcq	0.823	0.721	0.760	0.840	
TC	0.534	0.418	0.423	0.500	0.906

*TA, Technological Acquisition; RD, Research and Development; InInvs, Innovation Investment; ADI, Attitude toward Digital Innovation; TC, Technological Culture.*

### Structural Model

**FIGURE 3:**
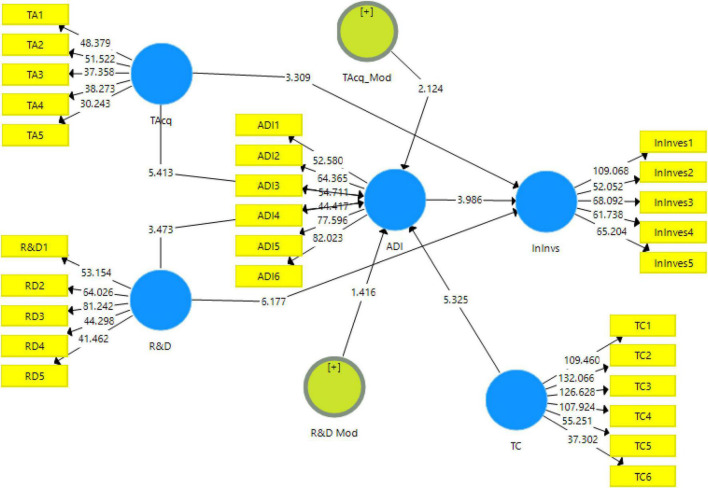
Output of structural model bootstrapping. TAcq= Technological Acquisition, R&D = Research and Development, InInvs= Innovation Investment, ADI = Attitude towards Digital Innovation, TC = Technological Culture.

The measurement model (explained above) has been examined and the criteria have been met. Now, the structural model has to be examined to carry out the data analysis. This model helps to analyze the relationship between the understudy variables by using partial least squares structural equation modeling (PLS-SEM) bootstrapping model (shown in Figure 3). The coefficient and *p*-values present in the model explain the relationship between the variables.

The direct relationship between the variables of the study has been analyzed using 95% bias-corrected bootstrap. The results for the proposed hypotheses of the study can be seen in Table 5 along with path coefficients, *t*-statistics, standard error, and *p*-values. PLS-SEM algorithms and PLS-SEM bootstrapping have been used in order to examine the hypotheses of the study. The results are based on the original sample mean, standard deviation, t-statistics, and *p*-values. For the acceptance of a hypothesis, the value of *t*-statistics should be greater than 1.96 (Winship and Zhuo, 2020), and the *p*-value should be less than 0.050 (Andrade, 2019).

**TABLE 5:** The direct effects of the variable.

Paths	H	O	M	SD	T-Statistic	*P*-value	Results
TA → InInvs	H_1_	0.203	0.208	0.061	3.309	0.001***	***Accepted***
TA → ADI	H_2_	0.430	0.445	0.079	5.413	0.000***	***Accepted***
RD → InInvs	H_3_	0.397	0.394	0.064	6.177	0.000***	***Accepted***
RD → ADI	H_4_	0.189	0.183	0.054	3.473	0.000***	***Accepted***
ADI → InInvs	H_5_	0.262	0.261	0.066	3.986	0.000***	***Accepted***

***p < 0.001.

*H, Hypothesis; O, Original Sample; M, Sample Mean; SD, Standard Deviation; TA, Technological Acquisition; RD, Research and Development; InInvs, Innovation Investment; ADI, Attitude toward Digital Innovation; TC, Technological Culture. Bold shows the variables and mentioned test significance and relationship.*

The first hypothesis (H_1_) is accepted (*t* = 3.309, *p* < 0.05), indicating that technological acquisition has a positive impact on innovative investment. Likewise, the second hypothesis (H_2_) is accepted (*t* = 5.413, *p* < 0.05), indicating that technological acquisition has a positive impact on attitude toward digital innovation. The third hypothesis (H_3_) is also accepted (*t* = 6.177, *p* < 0.05), indicating that R&D expenditure has a positive impact on innovative investment. The fourth hypothesis (H_4_) is similarly accepted (*t* = 3.473, *p* < 0.05), indicating that R&D expenditure has a positive impact on attitude toward digital innovation. Lastly, the fifth hypothesis (H_5_) is accepted (*t* = 3.986, *p* < 0.05), indicating that attitude toward digital innovation has a positive impact on innovative investment. The details have been shown in Table 5.

The indirect effect of attitude toward digital innovation between technological acquisition and innovative investment and between R&D expenditure and innovation investment has been shown in Table 6. The result shows that the sixth hypothesis (H_6_) of the study is accepted (*t* = 3.619, *p* < 0.05), indicating that attitude toward digital innovation mediates the relationship of technological acquisition and innovative investment. Moreover, the seventh hypothesis (H_7_) is accepted (*t* = 2.353, *p* < 0.05), indicating that attitude toward digital innovation mediates the relationship of research and development expenditure and innovative investment.

**TABLE 6:** The indirect effects of the variable.

Paths	H	O	M	SD	T-Statistic	*P*-value	Results
TA → ADI → InInvs	H_6_	0.113	0.115	0.031	3.619	0.000***	***Accepted***
RD → ADI → InInvs	H_7_	0.050	0.049	0.021	2.353	0.019*	***Accepted***

***p< 0.001, *p< 0.05.

*H, Hypothesis; O, Original Sample; M, Sample Mean; SD, Standard Deviation; TA, Technological Acquisition; RD, Research and Development; InInvs, Innovation Investment; ADI, Attitude toward Digital Innovation. Bold shows the variables and mentioned test significance and relationship.*

The moderating effect of technological culture between technological acquisition and attitude toward digital innovation and between R&D expenditure and attitude toward digital innovation is shown in Table 7. The result shows that the eighth hypothesis (H_8_) is accepted (*t* = 2.124, *p* < 0.05), indicating that technological culture moderates the relationship between technological acquisition and attitude toward digital innovation. However, the result shows that the ninth hypothesis (H_9_) of the study is rejected (*t* = 1.416, *p* > 0.05), indicating that technological culture does not moderate the relationship between research and development expenditure and attitude toward digital innovation.

**TABLE 7:** The moderating effects of the variable.

Paths	H	O	M	SD	T-statistic	*P*-value	Results
TA Mod → ADI	H_8_	–0.155	–0.137	0.073	2.124	0.034*	***Accepted***
RDMod → ADI	H_9_	–0.079	–0.091	0.056	1.416	0.157	***Rejected***

*p < 0.05.

*H, Hypothesis; O, Original Sample; M, Sample Mean; SD, Standard Deviation; TA Mod, Technological Acquisition Moderating effect; RD Mod, Research and Development Moderating Effect; ADI, Attitude toward Digital Innovation. Bold shows the variables and mentioned test significance and relationship.*

## Discussion

This research focused on certain gaps in previous research in the field of digital innovation regarding corporate sector organizations. Initially, some direct relationships identifying the role of technological acquisition and R&D expenditures in innovative investments were analyzed. Some direct relationships were also tested beginning with technological acquisitions and R&D expenditures on developing attitudes toward digital innovation in the organizations. This study also evaluated the mediating effects of attitude toward digital innovations between independent and dependent variables of the study, i.e., technological acquisitions and R&D expenditures and innovative investment. Some regulating roles of technological culture between technological acquisitions and R&D expenditures were also analyzed in this study, directing the attitudes toward digital innovation. This research produced some significant results which could contribute to the adoption of technological innovation culture in organizations. It can thus help devise proper and goal-directed investments for innovations with the help of acquiring useful technology and technological equipments and direct expenses for the R&D of the organizations. This could lead to optimized recommendations for organizations in the corporate sector in China.

The direct relationships were investigated by proposed hypothesis in this study. The first hypotheses was accepted, showing the significant role of technological acquisition in directing innovative investment. The second hypotheses also proved significant, indicating a significant role of technological acquisition on attitude toward digital innovation. The possible reasoning for these results lies in the nature of adopting such technology, which is crucial in the transformation toward the digital world of innovation. Directing the investments for innovation and developing attitudes among the people for digital innovation is strongly influenced by the acquisition of the technology. With the acquisition of technology, these objectives are merely impossible. This can be proven by many previous researches in the same field (Conte and Vivarelli, 2014).

The third and fourth hypothesis tested the relationships of R&D expenditures in directing the innovative investment and attitude toward digital innovation. Both hypotheses showed the significant relationship of these factors, indicating that spending in R&D could lead to direct investments for innovations in the digital transformation of the corporate sector organizations. This also indicated that spending in the R&D of organizations could lead to the development of attitudes of the people toward digital innovations. Similar results were also found to be significant in some of the previous research (Cao et al., 2021). The fifth hypothesis of this research evaluated the relationship of attitude toward digital innovation, directing the process of innovative investment. This also proved significant as attitudes of people direct them to do certain actions (Abu Bakar et al., 2020).

The indirect relationships were also studied in this research and indicated the roles of mediators and moderators. The mediating role of attitude toward digital innovation was accepted, indicating a significant mediating role between technological acquisitions and innovative investments and between R&D expenditures and innovative investments. Although the direct relationship was significant, it also showed that attitudes could be a helping hand in enhancing the relationships of these direct relationships. The mediator proved its significance, and it could be drawn from the results that, in future studies it would be again helpful in mediating the relationships for organizational transformations toward financial management. This study confirms the mediating impact of digital innovation by previous research (Khin and Ho, 2019).

The last two hypotheses of this research evaluated the moderating role of technological culture and gave similar and significant results to the previous researchers (Khin and Ho, 2019). The eighth hypothesis was accepted, indicating that the relationship of technological acquisition directing the attitude toward digital innovation was also moderated by technological culture. Meanwhile technological culture could not moderate the relationship of R&D expenditure on directing the attitude toward digital innovation. The direct relationship showed significant acceptance as R&D spending did not have any effect of an external regulator. While technological acquisition was moderated by the technological culture directing the attitude toward digital innovation, there was not much research in the past indicating the moderating role of technological culture. Despite this, a few presented a significant moderating role in technological culture (Khin and Ho, 2019).

## Conclusion

Examining the key drivers of attitude toward digital innovation and its overall impact on innovative investment is crucial to the R&D departments of IT firms. This is because these firms provide digital solutions to other industries, making them digitalize and influencing other industries to further innovate, thereby bringing more advanced technology to the market. Therefore, the current study investigated the role of technological acquisition and R&D expenditure in innovative investment. The study was conducted with the employees of the R&D department in the corporate sector of China. The study revealed that technological acquisition and R&D expenditure significantly and positively impacted innovative investment and attitude toward digital innovation. The study also found that attitude toward digital innovation among the employees positively and significantly impact innovative investment. Moreover, attitude toward digital innovation acts as a partial mediator between technological acquisition and innovative investment and between R&D expenditure and innovative investment. Furthermore, technological culture moderated the relationship between technological acquisition and innovative investment, but did not moderate the relationship between R&D expenditure and innovative investment.

## Theoretical Contributions

The study fills the gap in the literature by providing a comprehensive model that relates the R&D expenditure and technology acquisition to the innovative investments of the corporate sector. The present study has contributed to the literature of human resource development and organizational behavior by identifying the significant predictive roles of technological acquisition and R&D in the innovative investment of the organizations. Furthermore, the literature on organizational behavior has been extended by examining the significant mediating role of attitude toward digital innovation among the relationship between technological acquisition and innovative investment and between R&D and innovative investment. Similarly, different moderating variables had been mainstreamed in the past. However, the present study has introduced the moderating role of technological culture in understanding the impact of technological acquisition and R&D on attitude toward digital innovation. It has been statistically evidenced that technological culture significantly moderates the relationship of technological acquisition.

## Managerial Implications

Results of the study imply few connotations for the firms and their R&D departments. The R&D of the firms must develop a culture where state-of-the-art technological facilities are provided to the employees to augment their productivity and organizational performance. The use of technology should be made an important part of job design by providing training to the employees so that the technical skills of the employees can be polished and utilized for the betterment of the firms. Moreover, information technology-intensive firms must collaborate with giant players in the R&D sector so that the firms can utilize their human expertise to get better in technology. Furthermore, firms must retain and attract employees with high digital skills by developing new reward systems for encouraging technological culture.

## Limitations and Recommendations

Similar to other studies, the present study also has a few limitations. Firstly, the results for the current study were drawn from only one department of different firms of a country, affecting the generalizability of the findings. Therefore, future studies can be conducted in other regions and areas. Secondly, studies that would be conducted in the future can use a mediator, such as technological advancement, and a moderator, such as technological usefulness, in the current theoretical framework. Third, this study has used cross-sectional data with a quantitative approach. However, the interviews of the respondents could reveal certain other categories in the literature that could give better insights into these variables relationships. Hence, the mixed methods approach is supposed to give some interesting results.

## Data Availability Statement

The original contributions presented in the study are included in the article/supplementary material, further inquiries can be directed to the corresponding author/s.

## Author Contributions

ZW: conceptualization and writing the draft. AO: data collection and analysis. TG*:* editing and supervision. All authors contributed to the article and approved the submitted version.

## Conflict of Interest

The authors declare that the research was conducted in the absence of any commercial or financial relationships that could be construed as a potential conflict of interest.

## Publisher’s Note

All claims expressed in this article are solely those of the authors and do not necessarily represent those of their affiliated organizations, or those of the publisher, the editors and the reviewers. Any product that may be evaluated in this article, or claim that may be made by its manufacturer, is not guaranteed or endorsed by the publisher.
